# Eosinophile Cells in the Sputum in Asthma

**Published:** 1910-02-19

**Authors:** 


### Medicine.

### EOSINOPHILE CELLS IN THE SPUTUM IN ASTHMA.

The fact that coarsely granular eosinophile cells are frequently present in large numbers in the sputum of asthmatic patients may be of great service in the diagnosis of certain puzzling cases of chest disease. This has been well exemplified by Dr. John Hay, of Liverpool, who records the two following cases in which the eosinophile factor was of great assistance in determining the diagnosis of asthma in contra-distinction to tuberculosis of the lung.

### The First Case.

The first, Miss N., was a school teacher, aged thirty-six, of a nervous temperament, who gave a good family history. She was sent to Dr. Hay by her doctor in April 1907, who feared that she was attacked by phthisis. She had been healthy up to November 1906, when she had influenza, and had never been quite well since. She was troubled with a cough and some frothy expectoration, which contained no pus but occasionally a streak of blood. She expectorated at intervals all through the day and for an hour or two in the early morning. She had noticed some loss of weight. Her appetite was good, but she disliked fats and fruits, and could not eat bacon. There was no history of night sweats, and her temperature before noon was 99.4° F. This history was certainly suggestive. On examining the chest he found a few fine and medium-sized râles at the right apex in front; and though the breath sounds were unaltered, the percussion note at the outer half of the clavicle was slightly raised; it was also raised over the apex of the right upper lobe behind. The larynx was normal. With this history and these physical signs, he then felt fairly confident that he was dealing with a case of definite but early phthisis. When, however, he came to examine the sputum he failed to demonstrate any tubercle bacilli, and he was arrested by the unusual manner in which the leucocytes took up the Löffler's blue used as a counter-stain. The nuclei were well stained, but the cytoplasm appeared clear and unduly refractile. Accordingly he stained another specimen with eosin and methylene blue, and found that practically all the cells were coarsely granular eosinophiles, and on examining the unstained sputum microscopically it was possible to demonstrate the fibrin spirals, in which very many eosinophiles were entangled. Repeated examination of the sputum failed to reveal any tubercle bacilli, and he felt confident in concluding that he was dealing with a case of atypical asthma, complicated by some bronchitis, in which there was practically no dyspnœa, and in which the abnormal change in the lung had, at any rate at the time of his examination, been focussed in the right upper lobe. The treatment was accordingly directed to the relief of the asthmatic tendencies. He inquired recently from her doctor as to her progress, and heard from him that she was in fair health, and going about her work as usual.

The Second Case.

The second case in which the discovery of a marked eosinophilia in the sputum proved of good service in the clinching of the diagnosis was that of a medical man, æt. forty-two. He had been afflicted by a cough, which troubled him chiefly through the day, became worse on retiring for the night, and occasioned him some distress about 4 a.m. His speech was noticeably husky, and he was steadily losing weight. On examination Dr. Hay found the chest emphysematous; there was no dulness and no deficiency of movement; breath sounds were not altered, and there was no variation in the vocal resonance, but a few râles were to be heard at the left apex, and once or twice a rhonchus towards the base. Dr. Hunt reported favourably on the larynx; the cords were healthy, but there was some reddening of the arytenoids. There was no pyrexia, and the pulse-rate was normal. There was some tracheitis and possibly some bronchitis, but with such a history he could not exclude the possibility of pulmonary tubercle. The next step would have been to call in the aid of $x$-rays, but in the meantime he examined the sputum for tubercle bacilli, with negative results. On staining with eosin he found a marked eosinophilia. This strongly suggested to him that he was dealing with another case of atypical asthma—indeed, so atypical that the patient himself, a medical man, and an able one, had not suspected the nature of his malady. Another clinical fact corroborating the diagnosis of asthma was that the patient suffered from the presence of a few nasal polypi.

